# Triple lead cephalic versus subclavian vein approach in cardiac resynchronization therapy device implantation

**DOI:** 10.1038/s41598-018-35994-0

**Published:** 2018-12-07

**Authors:** Julia Vogler, Anne Geisler, Nils Gosau, Samer Hakmi, Stephan Willems, Tienush Rassaf, Reza Wakili, Elif Kaya

**Affiliations:** 1 Department of Electrophysiology, University Heart Center Hamburg, University Hospital Eppendorf, Hamburg, Germany; 2 Department of Cardiovascular Surgery, University Heart Center Hamburg, University Hospital Eppendorf, Hamburg, Germany; 3 Department of Cardiology and Vascular Medicine, West German Heart and Vascular Center, University Hospital Essen, Essen, Germany; 4 German Cardiovascular Research Center (DZHK), partner site: Munich Heart Alliance, Munich, Germany

## Abstract

Cardiac resynchronization therapy (CRT) device implantation is associated with severe complications including pneumo- and hemothorax. Data on a sole cephalic vein approach (sCV), potentially preventing these complications, are limited. The aim of our study was to compare a sole cSV with a subclavian vein approach (SV) in CRT implantations with respect to feasibility and safety. We performed a prospective cohort study enrolling twenty-four consecutive *de-novo* CRT implantations (group A) using a sCV at two centers. Fifty-four age-matched CRT patients implanted via the SV served (group B) as reference. Procedural success rate and complications were recorded during a follow-up of 4 weeks. All CRTs could be implanted in group A, with 91.7% using cephalic access alone. In group B, CRT implantation was successfully performed in 96.3%. Procedure and fluoroscopy duration were similar for both groups (sCV vs. SV: 119 ± 45 vs. 106 ± 31 minutes, 17 ± 9 vs 14 ± 9 minutes). Radiation dosage was higher in sCV group vs. SV (2984 ± 2370 vs. 1580 ± 1316 cGy*cm^2^; p = 0.001). There was no case of a pneumothorax in group of sCV, while two cases were observed using SV. Overall complication rate was similar (sCV: 13.0% vs. SV: 12.5%). *de-novo* CRT implantation using a triple cephalic vein approach is feasible. Procedure duration and complication rates were similar, while radiation dosage was higher in the sCV compared to the SV approach. Despite its feasibility in the clinical routine, controlled prospective studies with longer follow-up are required to elucidate a potential benefit with respect to lead longevity.

## Introduction

Cardiac resynchronization therapy (CRT) is an established and guideline recommended therapy in patients with congestive heart failure with severely reduced systolic function and left bundle branch block^1^. Although implantation techniques and left ventricular (LV) transvenous lead delivery systems have significantly improved, implantation of CRT devices is still associated with complications. These include pneumo- and hemothorax as severe acute complications. In analogy with other transvenous pacemaker or implantable cardioverter defibrillator (ICD) systems lead dysfunction is one of the most common problems during long-term follow-up.

Implantation techniques vary from center to operator and there is no consistent recommendation on a preferred vascular access site for placement of multiple pacing leads required in CRT therapy. Most centers use a combination of cephalic, subclavian, and/or axillary vein access. Since the introduction of peel away sheaths, subclavian vein puncture has been widely used as a primary access for insertion of pacemakers (PM) and ICDs leads due to its simplicity, speed, and high success rate^2–4^. Based on its low complication rate with respect to hemo- and pneumothoraces, a cephalic vein approach should be routinely intended as a primary vascular access. However, in CRT procedures, requiring a third (LV) lead, the majority of operators use an additional subclavian vein puncture in order to facilitate catheter manipulation for coronary sinus (CS) cannulation and to minimize interaction with the other leads reducing the risk of lead dislodgement^5^. Puncture of the subclavian vein carries a risk of pneumothorax (1–3%) or hemothorax compared to the cephalic vein preparation^6,7^. The subclavian access seems to be associated with lead failure and fracture due to the physical entrapment of the lead by the costoclavicular ligament and/ or the subclavius muscle (subclavian crush syndrome)^4,8–11^. Therefore, the option to position all three CRT leads via a single cephalic vein seems to be a potential alternative compared to an additional subclavian access for the LV lead in order to reduce the rate of severe complications. On the other side preparing the cephalic vein and positioning three leads through it requires training and can be technically more challenging potentially resulting in longer procedure times and more blood loss^2,4,12^. Little data exist on a sole cephalic vein approach with placement of all three leads via the cephalic vein in patients undergoing CRT pacemaker (CRT-P) and CRT defibrillator (CRT-D) implantation^5,13,14^.

The aim of our study was to compare a sole cephalic vein access on an intention-to-treat basis to a subclavian vein access in CRT implantation procedures with respect to feasibility and periprocedural outcome including complication rates.

## Methods

### Patient enrollment

We performed a prospective cohort study enrolling all consecutive patients between March 2016 and July 2017 undergoing a *de-novo* three-lead CRT-P or CRT-D implantation on an intention-to-treat basis using a sole cephalic vein (sCV) access for placement of all three leads. This group A represented the sCV approach. The study was performed at two university hospitals, the University Hospital Essen (Department of Cardiology and Vascular Medicine) and the University Heart Center Hamburg (Department of Electrophysiology).

A group n = 54 of age-matched patients served as a control group (group B). All matched patients in this group were implanted during the preceding 18 months in both centers (prior to the consecutive enrollment of the 24 patients in the cephalic vein group) using a subclavian vein access for placement of all three leads.

All patients enrolled in the study fulfilled standard criteria for CRT implantation according to the latest Guidelines of the European Society of Cardiology (ESC) and the ACA/AHA/HRS^1,15,16^ Patients with permanent atrial fibrillation, in whom only two leads (no atrial lead) were planned, and patients with CRT-upgrade procedures were excluded from the study.

All patients provided written informed consent and the study was performed in accordance with the relevant guidelines and regulations and the 1975 Declaration of Helsinki and was approved by the local ethics committee Protocol number 17-7701-BO University of University of Essen).

### Procedural workflows

Implant procedures were carried out under conscious sedation or general anesthesia. All patients received perioperative antibiotics.

#### Triple cephalic vein approach (group A)

For the cephalic vein access, incision was performed in the pectoral groove and venous dissection was performed to cannulate the cephalic vein. The first guidewire was inserted via a peripheral venous catheter (size: 18 Gauge) which was then exchanged to a 7 Fr sheath to put in the remaining two guidewires. The 7 F sheath was subsequently removed and a 9 Fr sheath was advanced over one of three guidewires to place the right ventricular (RV) lead (in CRT-P a 7 Fr sheath was used). The 9 Fr sheath was slit after the RV lead was in its final position. A 7 F sheath was then advanced to implant the right atrial (RA) lead followed by a 9 F sheath to place the LV lead. Each sheath was slit prior to adding a new one comparable to a standard subclavian vein approach. A hydrophilic guide wire was only used if we were not able advance the first standard guidewire through the cephalic vein. The sequence of lead placement in the cephalic vein group was RV lead, followed by the RA and finally LV lead.

Our triple cephalic vein approach thus differs from a recently described implantation technique by Hadjis *et al*.^5^. In the largest trial on a triple cephalic vein access (no control group) in CRT 150 of 171 consecutive patients who underwent de novo CRT implantation had all three lead successfully placed via cephalic vein. All implantations were performed by one operator who puts two long hydrophilic guidewires in the cephalic vein and an 11 Fr sheath alongside one of the two wires to advance the 9 Fr RV lead in CRT-D^5^. Two separate sheaths, 9 Fr and 7 Fr, for the right atrial (RA) and LV lead are advanced over the two retained wires after positioning of the RV lead and slitting of the 11Fr sheath. These 9 Fr and 7 Fr sheaths are not slit until the positioning of all three leads is complete in order to avoid lead interactions.

Contrast venography was not routinely performed unless no target vein could be identified or prepared. Subclavian vein puncture was performed in case of no available cephalic vein or a small cephalic vein prohibiting to implant three leads,

#### Subclavian vein approach (group B)

For the subclavian vein approach, direct puncture of the subclavian vein was performed (Seldinger technique) with separate puncture for each lead (3 punctures in total). Dilators and peel away sheaths were used to advance all pacing and defibrillation leads. Operators implanted RV lead (pacing or ICD) first, then the LV lead and finally the RA lead.

### Device implantation

The generator was positioned under the pectoral muscle in all CRT-D patients and over or under the pectoral muscle in CRT-P patients. Procedure duration was defined from the time of skin incision to the time of completion of skin closure, which is routinely recorded in the hospital computer system.

### Endpoints

The following procedural parameters were obtained: procedure and fluoroscopy duration, radiation exposure, success rates of the individual strategy as well as sensing and thresholds of each lead. Regarding safety we assessed the following periprocedural complications: pneumothorax or hemothorax with/without drainage, lead dislodgement requiring revision, CS dissection, pericardial effusion, pericardial tamponade, perforation, hematoma requiring evacuation, healing disorders, device/ lead infection, major bleeding events according to BRAC ≥3 criteria. Postprocedural complications as well as sensing and thresholds of each lead were recorded during a follow-up period of 4 weeks. Follow-up in both centers consisted of a routine device interrogation, a chest x-ray and a wound control on day 1 to 3 after implantation during the hospital stay and a device interrogation and wound control 4 weeks after implantation.

### Statistical analysis

All data were implemented into a database. Continuous variables are expressed as mean ± standard deviation in case of normal distribution, as median and interquartile range in the case of other distribution. Categorial variables are summarized as counts and percentage. The Mann-Whitney U test was used to compare continuous variables because variables were not normally distributed. Comparisons between the groups were performed using the Pearson’s chi square test or the Fisher’s exact test. An independent statistician performed all analyses using a statistical software package (IBM SPSS version 25, SPSS Inc. an IBM Company, Chicago, IL).

## Results

Between March 2016 and July 2017, a total of 78 patients undergoing *de-novo* CRT implantation were included in the study. Five patients (6.4%) received a CRT-P device and 73 (93.6%) a CRT-D device. Of these, 24 consecutive patients undergoing *de-novo* three lead CRT implant procedure via a sole cephalic vein access were prospectively enrolled into group A.

Fifty-four age-matched patients served as a control group. These patients were implanted during the 18 months preceding the enrollment of the 24 patients in the group A using a standard approach via the subclavian vein. Baseline characteristics of both groups were similar except for sex, body mass index (BMI) and LV function. The cephalic vein group A showed less male patients (n = 11; 45.8%) compared to the reference group B (n = 41; 75.9%). BMI and LV ejection fraction were both higher in group A compared to the subclavian vein group B (30 ± 5 kg/m^2^ versus 27 ± 5 kg/m^2^; 29 ± 6% versus 25 ± 7%). Dilative and ischemic cardiomyopathy were equally distributed in group A and B (50.0% versus 48.1% and 25.0% versus 31.5%; p = ns). Patient’s characteristics are summarized in Table 1.

**Table 1:** Patient baseline characteristics.

	Total, n = 78	Cephalic vein group, n = 24	Subclavian vein group, n = 54	p–value
Age [years]	68 ± 12	66 ± 12	68 ± 12	0.233
Male, n (%)	52 (66.7)	11 (45.8)	41 (75.9)	0.009
BMI [kg/m^2^]	28 ± 5	30 ± 5	27 ± 5	0.013
Diabetes, n (%)	27 (34.6)	9 (37.5)	18 (33.3)	0.721
Renal insufficiency, n (%)	42 (53.8)	7 (29.2)	35 (64.8)	0.004
Atrial fibrillation	35 (44.9)	5 (20.8)	30 (55.6)	0.004
Prior stroke, n (%)	11 (14.1)	1 (4.2)	10 (18.5)	0.158
Prior cardiac surgery	12 (15.4)	3 (12.5)	9 (16.5)	0.745
LVEF (%)	26 ± 7	29 ± 6	25 ± 7	0.029
Ischemic cardiomyopathy, n (%)	23 (29.5)	6 (25.0)	17 (31.5)	0.562
Dilative cardiomyopathy, n (%)	38 (48.7)	12 (50.0)	26 (48.1)	0.880

Data are presented as n (%) or mean ± standard deviation.

### Procedural results

CRT implantation was successfully performed in all 24 patients of the cephalic vein group A. Access via cephalic vein cut-down alone was accomplished in 22 patients. Additional puncture of the subclavian vein for LV lead placement due to a small cephalic vein was required in 1 of the 24 patients, implantation via the subclavian vein alone due to a missing cephalic vein was necessary in another patient.

In the subclavian vein group B, CRT implantation with respect to placement of the LV lead failed in 2 of the 54 patients during the first attempt (3.7%). In one patient, implantation failed due to a periprocedural CS dissection resulting in inability to implant the LV lead. The second implant failure was due to missing LV capture in the target vein (high epicardial LV threshold). The patient received surgical epicardial LV lead placement via left anterior mini-thoracotomy a couple weeks after failure of the transvenous approach resulting in effective biventricular pacing.

The majority of CRT implantations in our study was performed from the left side (100% in the cephalic vein group versus 92.6% in the subclavian vein group; p = ns). Quadripolar LV leads were used in 20 (83.3%) patients in the cephalic vein group and 39 (72.2%) patients in the subclavian vein group (p = ns). Mean procedure duration was slightly shorter when using a subclavian vein approach (119 ± . 45 versus 106 ± 31 minutes; p = 0.194) without reaching statistical significance. Fluoroscopy duration was similar in both groups (14 ± 9 versus 17 ± 9 minutes; p = 0.106) (Table 2). Only radiation exposure was significantly higher in the cephalic vein group (2984 ± 2370 versus 1580 ± 1316 cGy*cm^2^; p = 0.001).

**Table 2:** Procedural data.

	Cephalic vein group (n = 24)	Subclavian vein group (n = 54)	p-Value
CRT-D/ CRT-P, n (%)	21 (98.9)/3 (1.9)	53 (83.3)/1 (16.7)	0.029
Quadripolar lead, n (%)	20 (83.3)	39 (72.2)	0.291
Left-sided implantation, n (%)	24 (100)	50 (92.6)	0.306
Procedure duration [min]	119 ± 45	106 ± 31	0.194
Fluoroscopy duration [min]	16.5 ± 8.7	14.0 ± 8.8	0.106
Radiation exposure [cGy*cm^2^]	2985 ± 2369 [250; 9465]	1580 ± 1316 [258; 6219]	0.005
Dye solution [ml]	36 ± 14	58 ± 30	0.001

Data are presented as n (%) or mean ± standard deviation. Minimum and maximum (range) is displayed for radiation exposure.

Pacing thresholds of the LV lead (measured at 0.5 ms impulse duration) were similar in both groups at implantation (1.2 ± 0.6 V vs. 1.2 ± 0.5 V, p = 0.311), 24 hours after implantation (1.3 ± 0.7 V vs. 1.2 ± 0.6 V, p = 0.785) and 4 weeks after implantation (1.3 ± 0.7 V vs. 1.2 ± 0.5 V, p = 0.877). As expected, there was no difference in atrial and ventricular pacing thresholds. Long-term follow-up regarding lead performance was not available due to the short-term follow-up of 4 weeks.

### Periprocedural complications

Periprocedural complications occurred in 10 of 78 patients. There was no difference in the complication rate between patients with three leads implanted via cephalic vein versus those with subclavian vein puncture: In total, complications were noted in three patients (12.5%) in the cephalic vein group and seven patients (13.0%) in the subclavian vein group (Table 3). Lead dislodgement requiring lead repositioning or replacement was observed in two patients in each group. In addition, one patient in each group experienced a CS dissection during the attempt to implant the LV lead. No pneumothorax was observed in the cephalic vein group A, whereas two pneumothoraces (3.7%) occurred in the subclavian vein group B. Of the two pneumothorax cases one patient required a chest tube. Two further complications were noted in the subclavian group B: one hematoma requiring surgical evacuation in a patient with mechanical mitral and aortic valve prosthesis being on Vitamin K antagonist therapy, and one case of RV lead perforation without significant pericardial effusion resulting in lead repositioning.

**Table 3:** Outcome and complications in both patient groups.

	Cephalic vein group (n = 24)	Subclavian vein group (n = 54)	p-Value
Procedural success, n (%)	24 (100)	52 (96.3)	>0.999
Access failure, n (%)	2 (8.3)	0 (0)	0.09
Overall major complications, n (%)	3 (12.5)	7 (13.0)	>0.999
Lead dislodgement*	2 (8.3)	2 (3.7)	0.583
Coronary sinus dissection	1 (4.2)	1 (1.9)	0.523
Pneumothorax**	0 (0)	2 (3.7)	>0.999
Hematoma requiring surgical intervention	0 (0)	1 (1.9)	>0.999
Tamponade	0 (0)	0 (0)	n.a.
Infection	0 (0)	0 (0)	n.a.
Other***	0 (0)	1 (1.9)	>0.999

Data are presented as n (%).

Infection was defined as pocket infection and/or device-related endocarditis. *Lead dislodgement was one of the most common complications: One RA and one CS lead dislodgement was seen in each group. **With or without chest tube. ***One RV lead perforation requiring lead revision occurred in the subclavian vein group. RV lead perforation was recognized by severe chest pain and right ventricular exit block shortly after implantation. It was not associated with a relevant pericardial effusion. n.a.; non-applicable.

## Discussion

In our prospective analysis, *de-novo* CRT implantation could be accomplished in the majority of patients using a sole cephalic vein access. While procedure duration was similar, with a trend to be longer in the cephalic vein group, a sole cephalic vein approach was associated with a higher radiation dosage. Major complications rates did not differ between both groups.

### Procedural success

Our procedural results with an implantation success rate of 91.7% for an exclusive cephalic lead implantation are consistent to previous reports, which also evaluated a sole cephalic vein approach for CRT implantation^5,13,14^. With respect to the available and provided data, this approach seems feasible and underlies the fact, that a sole cephalic vein approach is possible in *de-novo* CRT implantations.

While some reports questioning a triple lead approach via the cephalic vein based on its often small diameter other studies describe that it is often not so much the “size” of the vein that predicts a successful implantation via the cephalic vein, but the cephalic anatomy^5,17^. Especially the course the vein takes to reach the subclavian vein, and the operator’s technique may play an important role^18,19^. Even a very small cephalic vein with an adequate course to the subclavian vein can be suitable to insert three leads, whereas an adequately sized vein with a supraclavicular vein may preclude placement of multiple leads.

These latter studies are in line with our data showing success rates >80%. However, the specific techniques facilitating implantation of all three leads through the cephalic vein seem still challenging. This is reflected by the procedural duration and radiation dosage in our study, which tended or were higher compared to the subclavian access group. In two cases, despite all efforts, we were not successful to implant all three leads via the cephalic vein due to a small cephalic vein in one patient and a missing cephalic vein in another patient. The higher radiation dosage in the cephalic vein group could be well explained by a higher BMI (30 ± 5 vs 27 ± 5 kg/m^2^; p = 0.013) and less optimized baseline fluoroscopy settings in the cephalic vein group. Fluoroscopy settings in group A (cephalic vein group) were in majority of cases 7.5 pictures/s instead of 3,5 pictures/s which was the mainly used setting in group B. In view of this different variables the observed difference in radiation dosage might be explained in part, but it has still to be considered that the sCV approach could be also associated with a higher radiation dosage. Our procedure duration in both groups correlates favorably with the reported implantation times of a 2013 EHRA survey (implantation time: 60–89 min in 36% of the centers, 90–119 min in 36%, <60 min in 17%, >119 min in 11%)^3^, which again stresses the point that cephalic vein cut-down might not be associated with longer procedure times, when performed by a trained cephalic vein operator.

### Periprocedural complications

The main rationale of our study was to show that a triple cephalic vein approach is feasible and able to reduce short-term complication rates in CRT implantation by mainly eliminating the risk of pneumo- and hemothorax with a modified and simple approach. Randomized CRT trials report a pneumothorax rate of up to 1.7%^20^. Pneumothorax was indeed the second most common major complication (3.7%) in the subclavian vein group, while this complication was absent in the cephalic vein group in our study. Despite no case of a pneumo- or hematothorax occurred in the sCV group, it has to be mentioned that the group size of the sCV group was smaller compared to the subclavian group. In total, the overall short-term complication rate did not differ between both groups (12.5% vs. 13.0%, p = ns). The overall complication rate in both groups appears to be high compared to a recently published study^5^, but is comparable to randomized clinical trials ranging from 8 up to 23%^20–26^. Looking at the complications in detail, we observed a trend towards increased rates of lead dislodgement and CS dissection in the cephalic vein group. While there was no statistical difference, these results pointing towards a higher lead dislodgement in the sCV group might be also due the limitation of low number of patients enrolled in the triple cephalic vein arm compared to the reference group being nearly twice as large. The results suggest that CRT related complications might occur during cephalic vein approach just as often as with subclavian vein puncture and might not as much dependent on the type of vascular access. Our higher lead dislodgement rate compared to other studies raises questions concerning our technique of slitting every sheath after lead placement which is different from the one described by Hadjis *et al*. However, with no intraoperative lead dislodgement an association with our technique of slitting sheaths seems unlikely.

### Mid-term Performance and follow up

In our analysis, atrial and ventricular pacing thresholds were similar for the cephalic and subclavian vein group. The follow-up period was yet too short to evaluate whether a sole cephalic vein access may offer a long-term benefit in biventricular pacing by reducing the rate of lead failure associated with subclavian crush syndrome. Axillary and subclavian vein puncture have been shown to be independent predictors of lead failure in a multicenter study of 3169 Fidelis leads, when compared with cephalic vein access^9^. The existing data on the relationship of the type of vascular access and the rate of lead failure are inconsistent. A recently published study revealed that the use of axillary vein puncture, not cephalic vein cut-down, independently predicted a lower risk of lead failure compared with subclavian vein puncture underlying the hypothesis of subclavia-associated lead failure^2^.

Large prospective trials comparing axillary, subclavian and cephalic vein access for CRT implantation are necessary to evaluate whether a triple cephalic vein approach is associated with a lower risk of long-term complications. As mentioned above, short-term complication rates were comparable between cephalic and subclavian vein access in our small cohort. Thus, according to our results and previously published trials the type of vascular access for CRT implantation seems to be at the operator’s discretion, when looking at these short-term periprocedural complication rates. If cephalic vein cut-down proves to be superior over subclavian vein puncture with regard to lead longevity or major complication rates in future trials, this approach might be recommended as a first line access in CRT implantation.

## Limitations

Our study is a small cohort study with a short-term follow-up of 4 weeks that allows to compare periprocedural complications between a triple cephalic vein access and a primary subclavian vein approach. No statement on a possible benefit regarding major complications or lead longevity with a cephalic vein access is possible based on this data set with limited statistical power. Only patients with cephalic vein cut-down were prospectively enrolled, whereas the control group consisted of patients previously implanted and was twice as large as the cephalic vein group. There was no randomization to a strategy of primary cephalic vein cut-down or primary subclavian vein puncture. In addition, the method of axillary vein puncture was not included in this analysis, which has also been reported to have certain advantages. Nevertheless, despite the limitations in statistical power and the non-randomized design this pilot study provides the basis for the feasibility of a triple cephalic vein CRT implantation concept. It has to be further tested in larger prospective randomized trials for its potential benefits.

## Conclusion

CRT implantation using a triple cephalic vein approach is feasible in the majority of patients undergoing *de-novo* three-lead CRT implantation. Overall complication rate did not differ between cephalic vs. subclavian vein approach in our small patient cohort. Additional larger randomized controlled multicenter studies with long-term follow-up are required to evaluate whether there is a potential benefit of the triple cephalic technique as a first line approach in patients undergoing CRT with regard to safety and lead longevity.

## Electronic supplementary material

Strobe

## Data Availability

For accessing additional data the senior author can be contacted with respect to specific request.
