# Supplementary material for: Triple lead cephalic versus subclavian vein approach in cardiac resynchronization therapy device implantation
Source: Sci Rep. 2018 Dec 7;8:17709. doi: 10.1038/s41598-018-35994-0 (PMC6286359; doi:10.1038/s41598-018-35994-0)
Supplement: Supplementary file 1 — Strobe [file 41598_2018_35994_MOESM1_ESM.pdf]

STROBE Statement—Checklist of items that should be included in reports of ***cohort studies***

	Item No	Recommendation
**Title and abstract**	1	(*a*) Indicate the study's design with a commonly used term in the title or the abstract<br><br>*Abstract: Section Methods: "We performed a prospective cohort study…"*
		(*b*) Provide in the abstract an informative and balanced summary of what was done and what was found<br><br>*An informative and balanced summary of what we did is given on page 2 (abstract)*
**Introduction**		
Background/rationale	2	Explain the scientific background and rationale for the investigation being reported<br><br>*Abstract page 2, introduction page 4, 2nd paragraph*
Objectives	3	State specific objectives, including any prespecified hypotheses<br><br>*Introduction, page 5, 2nd paragraph*
**Methods**		
Study design	4	Present key elements of study design early in the paper<br><br>*Methods, page 5, 1st and 2nd paragraph*
Setting	5	Describe the setting, locations, and relevant dates, including periods of recruitment, exposure, follow-up, and data collection<br><br>*Methods, page 5, patient enrollment 1-4. paragraph*<br>*Methods, page 7, endpoints (3rd paragraph)*
Participants	6	(*a*) Give the eligibility criteria, and the sources and methods of selection of participants. Describe methods of follow-up<br><br>*Methods: Patient enrollment, page 5,3rd paragraph. Patients were seen in the outpatient clinic for their routine 4-week follow-up. No further follow-ups were performed within this study.*
		(*b*) For matched studies, give matching criteria and number of exposed and unexposed<br>*methods, page 5, 3rd paragraph (age-matched)*
Variables	7	Clearly define all outcomes, exposures, predictors, potential confounders, and effect modifiers. Give diagnostic criteria, if applicable<br><br>*Methods, page 7, endpoints (3rd paragraph, page 7)*
Data sources/ measurement	8*	For each variable of interest, give sources of data and details of methods of assessment (measurement). Describe comparability of assessment methods if there is more than one group<br><br>*Methods, page 6, 2.-4. paragraph, page 7, 1.-2. paragraph, page 7, 3rd paragraph, last sentence; statistical analysis, page 7.*<br>*Consecutive patient enrollment (methods, page 5, 1st paragraph), age-matched historic control*
Bias	9	Describe any efforts to address potential sources of bias

		Methods, patient enrollment, page 5, 4th paragraph
Study size	10	Explain how the study size was arrived at<br><br>line 93-101
Quantitative variables	11	Explain how quantitative variables were handled in the analyses. If applicable, describe which groupings were chosen and why<br><br>*Statistical analysis, page 7*
Statistical methods	12	(*a*) Describe all statistical methods, including those used to control for confounding<br><br>*Statistical analysis, page 7*
		(*b*) Describe any methods used to examine subgroups and interactions<br><br>*No subgroup analysis was performed.*
		(*c*) Explain how missing data were addressed<br><br>*There were no missing data within this short follow-up period.*
		(*d*) If applicable, explain how loss to follow-up was addressed<br>*Not applicable.*
		(*e*) Describe any sensitivity analyses<br>*Not applicable.*
**Results**		
Participants	13*	(a) Report numbers of individuals at each stage of study—eg numbers potentially eligible, examined for eligibility, confirmed eligible, included in the study, completing follow-up, and analysed<br><br>*Results, page 8, 1st paragraph, 3rd paragraph*
		(b) Give reasons for non-participation at each stage<br>*Not applicable.*
		(c) Consider use of a flow diagram<br>*Not applicable based on study design.*
Descriptive data	14*	(a) Give characteristics of study participants (eg demographic, clinical, social) and information on exposures and potential confounders<br><br>*- Results, page 8, 1.-3. paragraph*<br>*- Table 1, patient baseline characteristics*
		(b) Indicate number of participants with missing data for each variable of interest<br><br>*No missing data.*
		(c) Summarise follow-up time (eg, average and total amount)<br><br>*The follow-up time for each participant was 4 weeks after CRT-implantation. Therefore, we do not provide an average and total amount of follow-up time. Methods, endpoints, last sentence.*
Outcome data	15*	Report numbers of outcome events or summary measures over time<br><br>*Results, periprocedural complication, page 9, 3rd paragraph (complications)*<br>*Results, procedural results, page 8, 3.-5. paragraph and page 9, 1.-2. paragraph*<br>*Table 2 and table 3*

Main results	16	(*a*) Give unadjusted estimates and, if applicable, confounder-adjusted estimates and their precision (eg, 95% confidence interval). Make clear which confounders were adjusted for and why they were included<br>*Not applicable.*
		(*b*) Report category boundaries when continuous variables were categorized<br>*Not applicable.*
		(*c*) If relevant, consider translating estimates of relative risk into absolute risk for a meaningful time period<br>*Not relevant*
Other analyses	17	Report other analyses done—eg analyses of subgroups and interactions, and sensitivity analyses<br>*Not applicable.*
**Discussion**		
Key results	18	Summarise key results with reference to study objectives<br>*Discussion, page 10, 1.-2. paragraph*
Limitations	19	Discuss limitations of the study, taking into account sources of potential bias or imprecision. Discuss both direction and magnitude of any potential bias<br>*Limitations, page 12*
Interpretation	20	Give a cautious overall interpretation of results considering objectives, limitations, multiplicity of analyses, results from similar studies, and other relevant evidence<br>*Discussion, page 12, 2nd paragraph*
Generalisability	21	Discuss the generalisability (external validity) of the study results<br>*Discussion, page 12, 2nd paragraph, last sentence*<br>*Conclusion, page 13*
**Other information**		
Funding	22	Give the source of funding and the role of the funders for the present study and, if applicable, for the original study on which the present article is based<br>*No disclosures. No funding.*

*Give information separately for exposed and unexposed groups.

Note: An Explanation and Elaboration article discusses each checklist item and gives methodological background and published examples of transparent reporting. The STROBE checklist is best used in conjunction with this article (freely available on the Web sites of PLoS Medicine at http://www.plosmedicine.org/, Annals of Internal Medicine at http://www.annals.org/, and Epidemiology at http://www.epidem.com/). Information on the STROBE Initiative is available at http://www.strobe-statement.org.
